# Association between physical activity, multimorbidity, self-rated health and functional limitation in the Spanish population

**DOI:** 10.1186/1471-2458-14-1170

**Published:** 2014-11-17

**Authors:** Cristina Cimarras-Otal, Amaia Calderón-Larrañaga, Beatriz Poblador-Plou, Francisca González-Rubio, Luis A Gimeno-Feliu, José L Arjol-Serrano, Alexandra Prados-Torres

**Affiliations:** GIMACES (E02) Research Group, San Jorge University, Autovía A-23 Zaragoza-Huesca Km. 299 50.830, Villanueva de Gállego, Spain; EpiChron Research Group on Chronic Diseases, Aragón Health Sciences Institute (IACS), IIS Aragón, Miguel Servet University Hospital, Paseo Isabel La Católica 1-3, 50009 Zaragoza, Spain; University of Zaragoza, C/Domingo Miral s/n, 50009 Zaragoza, Spain; Red de Investigación en Servicios de Salud en Enfermedades Crónicas (REDISSEC), Carlos III Health Institute, C/Sinesio Delgado 4, 28029 Madrid, Spain; South Delicias Health Centre, Aragon Health Service (SALUD), C/de Manuel Dronda 1, 50009 Zaragoza, Spain; San Pablo Health Centre, Aragon Health Service (SALUD), C/Aguadores 7, 50003 Zaragoza, Spain

**Keywords:** Physical activity, Multimorbidity, Self-rated health, Activity limitations, European Health Interview Survey

## Abstract

**Background:**

Physical activity (PA) has been shown to improve the general health of patients with chronic diseases and to prevent the onset of such conditions. However, the association between multimorbidity and PA has not been investigated in detail, and recent studies of this topic yield dissenting results. The objective of this study was to examine whether PA levels were associated with multimorbidity, self-rated health and functional limitation.

**Methods:**

This was a cross-sectional study based on data from the 2009 European Health Interview Survey for Spain. The sample population included 22,190 adults over 15 years of age. The independent variables were multimorbidity (measured as the number of chronic diseases), activity limitations, and self-rated health status. The dependent variable was PA level, measured as a) a continuous variable in metabolic equivalents (METs) and b) a dichotomous variable based on international recommendations (</≥500 MET-minutes per week). The associations between the dependent and independent variables were evaluated across sex and age groups (16–24, 25–44, 45–64, 65–74, >74 years), using multivariate linear and logistic regression models that were adjusted for age, educational level and employment status.

**Results:**

An inverse association was found between PA and multimorbidity among older males and young females between 16–24 years. This negative association was also observed among males aged 25–44 years when analysing PA as a dichotomous variable. Self-rated health status was directly related to the achievement of minimum PA levels among middle-aged and older individuals, but the opposite happened among the youngest group of females. Significant associations between the existence of activity limitations and the performance of lower volumes of PA were consistently observed among subjects over 44 years.

**Conclusions:**

There is an inverse association between multimorbidity and PA in the youngest and oldest age groups. In addition, both low self-rated health status and the presence of functional limitations were related to lower PA in most of the examined population groups. These features should be considered in the design and implementation of community-based approaches to promoting PA, if further corroborated in longitudinal studies.

## Background

One of the main objectives of national health systems in developed countries is the prevention of chronic disease. Among the lifestyle strategies for improving the health of individuals with chronic disease, the promotion of physical activity (PA) is extensively supported in the published literature [1–3]. Regular PA contributes to the primary and secondary prevention of several chronic diseases and is associated with a reduced risk of premature death [4]. There appears to be a graded linear relationship between volume of PA and health status in which the most physically active individuals have the lowest health risks [4]. It has been demonstrated that relative to sedentary individuals, more active males and females show lower rates of all-cause mortality, coronary heart disease, high blood pressure, stroke, type 2 diabetes, metabolic syndrome, colon cancer, breast cancer, and depression [1–6]. PA also contributes to overall quality of life by increasing individuals’ strength, ability to perform daily chores and participate in social interactions, mobility, cognitive performance, and life expectancy [2].

Although PA appears to be effective for the prevention of chronic disease and premature death, it remains uncertain exactly what the optimal volume of PA is in terms of frequency, duration, and intensity of PA, and what is the minimum volume of PA required to obtain health benefits. In particular, there is debate regarding the effects of PA intensity (e.g., moderate vs. vigorous) on health status. It is difficult to use extant knowledge to derive a precise single expression for the PA level associated with improved health because published reports differ with respect to the type of PA, the conditions under which PA is performed, and its measurement units [1]. Several organisations, such as the American College of Sports Medicine (ACSM), the American Heart Association (AHA), and the US Department of Health and Human Services have attempted to summarise the recommendations regarding the most appropriate volume of PA for health maintenance and the prevention of chronic disease in the population [1, 6, 7]. These organisations have characterised aerobic activities of various types and intensities in terms of a single measure of PA, the metabolic equivalent (MET). The quantity of moderate and vigorous PA that has been associated with significantly lower rates of disease and/or improvements in biomarker and fitness levels has changed over time; however, in 2011, the ACSM suggested a target range of 500–1000 MET-minutes per week [6].

An issue that requires further investigation is the relationship between PA and multimorbidity, defined as the presence of multiple chronic diseases [8]. The number of individuals with multimorbidity is rapidly increasing due not only to environmental and medical advances that have preserved and extended lives, but also to a continued growth in the proportion of older individuals around the world [9]. Recent findings revealed an inverse association between PA and multimorbidity among older males but not among older females [10]; yet results on this topic remain inconclusive [11].

In contrast, the positive association between better self-rated health and PA levels and the negative association between the latter and the existence of functional limitations have been consistently acknowledged in previous studies [11–15].

The purpose of this study was to examine the association between levels of PA and multimorbidity, self-rated health and functional limitations for different age- and sex-based groups of Spanish subjects.

## Methods

### Data source

This investigation was based on the 2009 European Health Interview Survey, a five-year survey carried out in 18 countries of the European Union. We only had access to the Spanish survey (http://www.ine.es/en/metodologia/t15/t153042009cues_en.pdf) which was conducted by the National Statistics Institute (INE) [16]. The main objective of this survey is to collect data regarding individuals’ health status, lifestyle, and use of healthcare services by means of a three-stage, stratified sampling strategy. The first-stage units are the census tracts and the second-stage units correspond to family residential zones. In the second stage of the sampling process, all households of a given residential zone are considered. In the third stage of this process, one adult (i.e. ≥16 years) per household is randomly chosen to complete an individual questionnaire. Whenever the randomly chosen household/respondent failed, the interviewer could replace the household by the first available valid reserve dwelling.

In the case of Spain, out of the total number of incumbent households to be interviewed, 64.1% were actually surveyed (i.e. 73.3% of surveyable households) and 32.4% were replaced by a reserve, thus increasing the effective total sample to 96.5% of the theoretical sample. In other words, approximately 23,000 households distributed across 1,927 census tracts -representative at both national and regional levels- were selected, and a total of 22,190 computer-assisted personal interviews were conducted.

For analysis purposes, the sample was divided into five age groups (16–24, 25–44, 45–64, 65–74, >74 years) and by sex in order to capture the potential interactions of these variables with the study factors.

### Independent variables

Multimorbidity, which is defined as the co-occurrence of two or more diseases within a single individual [8], was determined from self-reported data regarding the following diseases included in the survey: asthma, chronic bronchitis, cardiac infarction, coronary heart disease, hypertension, stroke, rheumatoid arthritis, osteoporosis, chronic back/neck pain, diabetes, allergies, gastric ulcers, cirrhosis, cancer, frequent headaches, urinary incontinence, chronic anxiety, chronic depression, other mental disorders, and permanent accident injuries. Only health problems diagnosed by a physician and experienced during the preceding 12 months were considered for analysis. The variable multimorbidity was classified into four categories (0, 1, 2 and ≥3 diseases). When defining multimorbidity, the threshold of three or more concomitant disease entities seems to provide greater specificity than only two or more conditions [17].

To gather information regarding long-term activity limitations, interviewees were asked whether they had been “severely limited”, “limited but not severely” or “not limited” in the performance of routine activities due to a health problem for at least the last six months. For the sake of simplicity this variable was incorporated into the models as a dichotomous one indicating either the absence or presence of self-reported activity limitations. When comparing this variable with data on Activities of Daily Living (ADL) and/or Instrumental Activities of Daily Living (IADL) limitations gathered in the same survey, we found that the former is a somewhat more sensitive measure of functional limitations. That is, an important number of subjects showing no difficulty in carrying out the different ADL/IADL were classified as being limited according to the variable employed in this study.

Data on subjects’ self-rating of their general health during the last 12 months were also obtained from the survey. The five original categories used in the survey were grouped into the following two categories for the purpose of this study: very poor to normal and good to very good.

Finally, different socio-demographic characteristics, such as age, sex, educational level, and employment status, were included in the models as covariates to account for the potential confounding effects of these variables.

### Dependent variable

In the survey, individuals were asked to indicate any vigorous, moderate, or light PA they had performed in the previous seven days during the course of leisure/entertainment activities, household chores, or work-related pursuits. The calculation of the weekly leisure time devoted to PA for each surveyed individual was based on METs, which reflect estimates of the ratio of energy expended during a certain PA to energy expended while sitting quietly. The number of weekly hours and minutes a subject dedicated to each type of activity was multiplied by the MET value assigned to that activity based on the International Physical Activity Questionnaire (IPAQ) criteria [18]. More specifically, activities were classified as low (3.3 METs), moderate (4 METs), or vigorous (8 METs) PAs. Based on the current ACSM recommendations [1], significantly lower rates of disease and improvements in biomarker and fitness levels are associated with 500 to 1000 MET-minutes per week of moderate to vigorous PA [6]. Using this criterion, we created a dichotomous variable to reflect whether subjects achieved this minimum threshold of PA or not (< or ≥500 MET-minutes per week).

### Statistical analyses

Pearson's chi-squared test was applied for the identification of gender differences in the distribution of the study variables. Linear multivariate regressions were employed when studying the dependent variable as continuous (i.e. total MET-hours per week), and logistic regressions were used to analyse the dependent variable as dichotomous (i.e. < or ≥500 MET-minutes per week).

All covariates (age, educational level, and employment status) were included as control variables in each model. The analyses were conducted with the STATA software, version 12. Statistical significance was accepted for P-values <0.05.

## Results

The study sample was composed of 22,190 individuals (45.28% males and 54.72% females). The prevalence of multimorbidity, lower self-rated general health and functional limitation was significantly higher among females of almost all age groups (Table 1). Regarding PA, males showed a higher probability of complying with the minimum PA recommended by the ACSM both in the youngest and oldest age groups (Table 1).

**Table 1:** **Characteristics of the study population**

	16-24 years				25-44 years				45-64 years				65-74 years				>74 years			
	Males		Females		Males		Females		Males		Females		Males		Females		Males		Females	
	N = 824		N = 813		N = 3594		N = 3837		N = 3298		N = 3797		N = 1209		N = 1641		N = 1122		N = 2055	
	N	(%)	N	(%)	N	(%)	N	(%)	N	(%)	N	(%)	N	(%)	N	(%)	N	(%)	N	(%)
**Number of chronic diseases**																				
0	602	73.06	567	69.74	2340	65.11*	2177	56.74*	1551	47.03*	1318	34.71*	337	27.87*	252	15.36*	193	17.20*	197	9.59*
1	161	19.54	142	17.47	824	22.93	860	22.41	806	24.44*	828	21.81*	312	25.81*	267	16.27*	287	25.58*	339	16.50*
2	42	5.10*	71	8.73*	277	7.71*	399	10.40*	437	13.25	558	14.70	248	20.51*	280	17.06*	239	21.30*	367	17.86*
≥3	19	2.31	33	4.06	153	4.26*	401	10.45*	504	15.28*	1093	28.79*	312	25.81*	842	51.31*	403	35.92*	1152	56.06*
**Self-rated general health**																				
Very poor to normal	54	6.55*	87	10.70*	466	12.97*	705	18.37*	993	30.11*	1449	38.16*	560	46.32*	993	60.51*	671	59.80*	1,463	71.19*
Good to very good	770	93.45*	726	89.30*	3128	87.03*	3132	81.63*	2305	69.89*	2348	61.84*	649	53.68*	648	39.49*	451	40.20*	592	28.81*
**Long-term activity limitations**																				
Not limited	771	93.57	740	91.02	3170	88.20*	3258	84.91*	2543	77.11*	2623	69.08*	778	64.35*	836	50.94*	500	44.56*	670	32.60*
Limited	53	6.43	73	8.98	424	11.8*0	579	15.09*	755	22.89*	1174	30.92*	431	35.65*	805	49.06*	622	55.44*	1385	67.40*
**Educational level**																				
Lowest^1^	23	2.79	15	1.85	111	3.09	105	2.74	345	10.46*	484	12.75*	372	30.77*	715	43.57*	552	49.20*	1166	56.74*
Low^2^	459	55.70*	398	48.95*	1368	38.06*	1164	30.34*	1468	44.51	1759	46.33	531	43.92	702	42.78	410	36.54	733	35.67
Average^3^	292	35.44	321	39.48	1329	36.98	1371	35.73	898	27.23*	910	23.97*	174	14.39*	140	8.53*	79	7.04*	86	4.18*
High^4^	49	5.95*	79	9.72*	785	21.84*	1197	31.20*	582	17.65	641	16.88	130	10.75	81	4.94	78	6.95*	66	3.21*
Unknown/ No response	1	0.12	0	0.00	1	0.03	0	0	5	0.15	3	0.08	2	0.17	3	0.18	3	0.27	4	0.19
**Employment status**																				
Not working^5^	605	73.42	615	75.65	751	20.90*	1334	34.77*	1088	32.99*	1965	51.75*	1157	95.70*	1598	97.38*	1119	99.73	2050	99.76
Working	218	26.46	197	24.23	2841	79.05*	2501	65.18*	2205	66.86*	1832	48.25*	52	4.30*	41	2.50*	3	0.27	5	0.24
No response	1	0.12	1	0.12	2	0.06	2	0.05	5	0.15	0	0.00	0	0.00	2	0.12	0	0.00	0	0.00
**Physical activity (≥500) METs)**																				
No	123	14.93*	212	26.08*	780	21.70*	916	23.87*	770	23.35	901	23.73	281	23.24*	502	30.59*	409	36.45*	1036	50.41*
Yes	701	85.07*	601	73.92*	2814	78.30*	2921	76.13*	2528	76.65	2896	76.27	928	76.76*	1139	69.41*	713	63.55*	1019	49.59*

^1^Lowest: “Cannot read or write” or “Did not complete primary school”. ^2^Low: “Primary school or equivalent” or “Compulsory secondary education”. ^3^Average: “Baccalaureate degree” to “Higher-level vocational studies”. ^4^High: “University degree” or “Doctoral degree”. ^5^Not working: “Unemployed”, “Student, apprentice, or intern”, “Retired”, “Unable to work”, “Dedicated to housework”, or “Other”.

*Statistically significant differences between males and females for a given age group (p-value <0.05).

Tables 2, 3, 4, and 5 show the results for the two regression models. There was a clear correlation between multimorbidity and lower PA levels among older males over 74 and the youngest female group. The same occurred in middle-aged males with one single chronic disease (Table 2). In the model with PA as a dichotomous variable, this correlation was also observed among young males aged 25–44 years (Table 4). Among middle-aged and most of the older age groups, the variable of self-rated health status was directly related to the achievement of the minimum PA levels recommended by the ACSM (Tables 4 and 5). Surprisingly, among the youngest group of females, poorer self-ratings of health status were associated with the achievement of higher PA levels (Tables 3 and 5). In both regression models, statistically significant associations between activity limitations and the performance of lower volumes of PA were observed among middle-aged and older subjects.

**Table 2:** **Beta coefficients and p values from multinomial linear models, with MET-hours/week as the dependent variable, among males**

	16-24 years		25-44 years		45-64 years		65-74 years		>74 years	
**R squared**	0.085		0.031		0.052		0.046		0.115	
**Age**	-0.199	0.886	-1.188*	0.000	-0.204	0.487	-0.913	0.065	-0.930*	0.000
**Number of chronic diseases**										
0 (ref. cat.)										
1	-9.662	0.200	3.987	0.335	-12.880*	0.002	-5.244	0.184	-1.689	0.598
2	-10.428	0.443	2.413	0.716	-4.730	0.369	-2.560	0.560	-6.088	0.070
≥3	-13.208	0.522	1.503	0.868	-10.078	0.074	-5.754	0.206	-9.194*	0.006
**Self-rated general health**										
Very poor to normal (ref. cat.)										
Good to very good	-2.340	0.861	0.820	0.887	5.466	0.217	11.680*	0.001	10.715*	0.000
**Long-term activity limitations**										
Not limited (ref. cat.)										
Limited	-5.926	0.665	4.729	0.434	-13.628*	0.003	-8.056*	0.023	-9.316*	0.000
**Educational level**										
Lowest^1^ (ref. cat.)										
Low^2^	20.476	0.274	-3.545	0.720	-2.879	0.603	-0.181	0.957	-0.820	0.714
Average^3^	0.973	0.959	-11.237	0.259	-27.031*	0.000	3.952	0.399	0.476	0.908
High^4^	-6.451	0.770	-34.986*	0.001	-45.065*	0.000	-4.299	0.410	-2.493	0.558
**Employment**										
Not working^5^ (ref. cat.)										
Working	50.819*	0.000	34.516*	0.000	24.149*	0.000	6.498	0.372	-19.963	0.316

^1^Lowest: “Cannot read or write” or “Did not complete primary school”. ^2^Low: “Primary school or equivalent” or “Compulsory secondary education”. ^3^Average: “Baccalaureate degree” to “Higher-level vocational studies”. ^4^High: “University degree” or “Doctoral degree”. ^5^Not working: “Unemployed”, “Student, apprentice, or intern”, “Retired”, “Unable to work”, “Dedicated to housework”, or “Other”.

*Statistical significance (p-value <0.05).

**Table 3:** **Beta coefficients and p values from multinomial linear models, with MET-hours/week as the dependent variable, among females**

	16-24 years		25-44 years		45-64 years		65-74 years		>74 years	
**R squared**	0.044		0.015		0.017		0.032		0.082	
**Age**	1.758	0.071	-0.171	0.474	-0.356	0.106	-1.164*	0.009	-1.180*	0.000
**Number of chronic diseases**										
0 (ref. cat.)										
1	-3.391	0.539	5.468	0.097	-0.752	0.820	-1.461	0.754	-3.182	0.327
2	-0.906	0.906	6.846	0.132	4.754	0.220	3.341	0.478	-2.796	0.388
≥3	-22.862*	0.039	1.835	0.709	1.659	0.666	1.311	0.768	-3.791	0.213
**Self-rated general health**										
Very poor to normal (ref. cat.)										
Good to very good	-21.362*	0.004	-5.596	0.157	7.095*	0.030	-0.699	0.839	4.544*	0.038
**Long-term activity limitations**										
Not limited (ref. cat.)										
Limited	-7.233	0.356	-1.670	0.693	-1.161	0.724	-16.597*	0.000	-13.611*	0.000
**Educational level**										
Lowest^1^ (ref. cat.)										
Low^2^	14.692	0.358	11.835	0.152	6.004	0.117	-1.158	0.686	1.681	0.326
Average^3^	9.172	0.568	2.190	0.791	-2.874	0.512	6.263	0.210	-3.421	0.398
High^4^	-10.290	0.549	-11.234	0.181	-13.482*	0.005	-14.048*	0.028	-2.530	0.582
**Employment**										
Not working^5^ (ref. cat.)										
Working	15.311*	0.004	11.241*	0.000	13.737*	0.000	4.978	0.555	-3.074	0.849

^1^Lowest: “Cannot read or write” or “Did not complete primary school”. ^2^Low: “Primary school or equivalent” or “Compulsory secondary education”. ^3^Average: “Baccalaureate degree” to “Higher-level vocational studies”. ^4^High: “University degree” or “Doctoral degree”. ^5^Not working: “Unemployed”, “Student, apprentice, or intern”, “Retired”, “Unable to work”, “Dedicated to housework”, or “Other”.

*Statistical significance (p-value <0.05).

**Table 4:** **OR and 95% CI from multinomial logit models, with physical activity relative to the ACSM threshold as the dependent variable, among males**

	16-24 years			25-44 years			45-64 years			65-74 years			>74 years		
**Prob > chi squared**	0.118			0.000			0.000			0.000			0.000		
	**OR**	**95% CI**		**OR**	**95% CI**		**OR**	**95% CI**		**OR**	**95% CI**		**OR**	**95% CI**	
**Age**	0.945	0.862	1.035	0.989	0.974	1.003	1.008	0.993	1.024	0.984	0.938	1.032	0.937*	0.911	0.964
**Number of chronic diseases**															
0 (ref. cat.)															
1	0.896	0.549	1.462	1.007	0.825	1.230	0.979	0.793	1.208	1.053	0.697	1.590	0.916	0.576	1.455
2	1.004	0.414	2.432	0.976	0.714	1.334	1.238	0.936	1.636	1.104	0.705	1.730	0.644	0.402	1.031
≥3	0.740	0.233	2.350	0.660*	0.448	0.973	1.038	0.784	1.375	0.686	0.445	1.057	0.515*	0.329	0.807
**Self-rated general health**															
Very poor to normal (ref. cat.)															
Good to very good	1.592	0.739	3.431	1.106	0.848	1.443	1.550*	1.243	1.933	1.532*	1.089	2.157	1.886*	1.334	2.667
**Long-term activity limitations**															
Not limited (ref. cat.)															
Limited	0.767	0.342	1.716	0.856	0.649	1.129	0.691*	0.551	0.866	0.575*	0.414	0.797	0.405*	0.293	0.561
**Educational level**															
Lowest^1^ (ref. cat.)															
Low^2^	2.701*	1.031	7.073	1.094	0.706	1.693	1.010	0.765	1.332	0.775	0.562	1.068	1.038	0.777	1.386
Average^3^	2.852*	1.069	7.606	1.371	0.880	2.136	1.124	0.830	1.521	1.354	0.818	2.242	0.722	0.421	1.239
High^4^	1.724	0.539	5.509	1.685*	1.060	2.677	1.286	0.921	1.795	1.067	0.628	1.812	1.465	0.782	2.744
**Employment**															
Not working^5^ (ref. cat.)															
Working	1.253	0.769	2.042	1.140	0.936	1.388	0.780*	0.642	0.949	0.825	0.392	1.738	0.212	0.018	2.508

^1^Lowest: “Cannot read or write” or “Did not complete primary school”. ^2^Low: “Primary school or equivalent” or “Compulsory secondary education”. ^3^Average: “Baccalaureate degree” to “Higher-level vocational studies”. ^4^High: “University degree” or “Doctoral degree”. ^5^Not working: “Unemployed”, “Student, apprentice, or intern”, “Retired”, “Unable to work”, “Dedicated to housework”, or “Other”.

*Statistical significance (p-value <0.05).

**Table 5:** **OR and 95% CI from multinomial logit models, with physical activity relative to the ACSM threshold as the dependent variable, among females**

	16-24 years			25-44 years			45-64 years			65-74 years			>74 years		
**Prob > chi squared**	0.143			0.049			0.000			0.000			0.000		
	**OR**	**95% CI**		**OR**	**95% CI**		**OR**	**95% CI**		**OR**	**95% CI**		**OR**	**95% CI**	
**Age**	0.954	0.885	1.028	0.998	0.985	1.012	1.009	0.995	1.024	0.963*	0.928	0.999	0.918*	0.900	0.936
**Number of chronic diseases**															
0 (ref. cat.)															
1	1.182	0.759	1.842	0.967	0.802	1.166	1.151	0.924	1.434	0.942	0.621	1.429	1.047	0.710	1.542
2	0.851	0.474	1.527	1.137	0.873	1.482	1.236	0.956	1.598	0.945	0.622	1.434	1.269	0.861	1.870
≥3	0.416*	0.190	0.912	1.052	0.796	1.391	1.064	0.833	1.359	0.869	0.589	1.280	0.845	0.590	1.210
**Self-rated general health**															
Very poor to normal (ref. cat.)															
Good to very good	0.501*	0.269	0.936	1.081	0.865	1.350	1.526*	1.241	1.876	1.194	0.892	1.598	1.687*	1.307	2.177
**Long-term activity limitations**															
Not limited (ref. cat.)															
Limited	0.710	0.394	1.278	0.891	0.703	1.130	0.795*	0.648	0.975	0.523*	0.400	0.685	0.436*	0.345	0.552
**Educational level**															
Lowest^1^ (ref. cat.)															
Low^2^	1.379	0.441	4.308	1.888*	1.244	2.865	1.437*	1.145	1.804	1.295*	1.024	1.638	1.080	0.885	1.318
Average^3^	1.684	0.535	5.298	2.105*	1.383	3.202	1.517*	1.161	1.982	1.430	0.918	2.228	1.064	0.660	1.714
High^4^	1.223	0.359	4.168	2.146*	1.399	3.293	1.905*	1.404	2.585	0.911	0.534	1.555	0.956	0.552	1.655
**Employment**															
Not working^5^ (ref. cat.)															
Working	1.238	0.823	1.864	1.027	0.871	1.210	0.898	0.758	1.064	0.611	0.309	1.208	1.823	0.195	17.037

^1^Lowest: “Cannot read or write” or “Did not complete primary school”. ^2^Low: “Primary school or equivalent” or “Compulsory secondary education”. ^3^Average: “Baccalaureate degree” to “Higher-level vocational studies”. ^4^High: “University degree” or “Doctoral degree”. ^5^Not working: “Unemployed”, “Student, apprentice, or intern”, “Retired”, “Unable to work”, “Dedicated to housework”, or “Other”.

*Statistical significance (p-value <0.05).

Regarding the remaining covariates, age was inversely associated with PA performance, particularly among older male and female subjects. The educational level variable exhibited ambiguous results depending on the model used: specifically, an inverse association between educational level and PA was observed in the model with a continuous dependent variable among males between 25–64 years and females between 45–74 years (Tables 2 and 3), whereas a direct and gradual association between these variables was observed in the model with a dichotomous dependent variable among males between 25–44 years and among females between 25–64 years (Tables 4 and 5). With respect to employment status, being actively employed was directly related to PA volume in young and middle-aged subjects of both sexes (Tables 2 and 3). Among the group of middle-aged males, being actively employed was inversely associated with the achievement of minimum PA levels (Table 4), but this association was restricted to men of around 64 years old (analysis not shown).

## Discussion

We found that multimorbidity was inversely associated with PA in the youngest and oldest age groups after controlling for long-term activity limitations, self-perceived health status, age, sex, educational level, and employment status. Our results differ from those of Hudon et al. [11], who concluded that multimorbidity was not associated with PA levels for Canadian adults of either sex. Our findings partially coincide with those published in a recent German study that demonstrated an inverse association between PA and multimorbidity only in older males [10]. To the best of our knowledge, no prior studies reported an inverse association between multimorbidity and PA among younger subjects, which deserves further attention.

The different methods used to measure PA in each of the studies cited above might explain the dissimilar results of these investigations. The European Health Interview Survey measured PA based on the IPAQ questionnaire; this approach was similar to the methodology used in the aforementioned German study (which employed the Physical Activity Scale for the Elderly (PASE)). Both surveys asked subjects to provide the minutes of PA performed during the seven days prior to the completion of the questionnaire [19]. In contrast, Hudon et al. measured PA using the number of 20–30 minute sessions of leisure-related PA that subjects had engaged in during the preceding three months [11]. Other causes underlying the differences with previous studies could be related to the division of our study sample into narrower age groups, which could reveal otherwise unnoticed associations.

With respect to the variable of self-rated health, the study results support the conclusion that among middle-aged and older individuals, good to very good self-rated health is directly related to the achievement of the minimum levels of PA recommended by the ACSM. These results are consistent with the findings of studies conducted in Korea [12] and Canada [11] as well as the conclusions of a systematic review by Bize et al. published in 2007 [15]. However, the opposite was observed among the group of very young females; in other words, among these subjects, poorer self-ratings of health status were associated with higher PA levels. Due to the cross-sectional design of the present study, it is not possible to establish the direction or the eventual causality of this correlation. It is most likely that young females with poor health status could be more compelled to increase their PA levels.

As was expected, long-term activity limitations were associated with lower PA levels among middle-aged and older males and females [11, 14]. Future longitudinal studies are required to better explain the temporal link between these variables and the lack of association observed among younger subjects.

Age was inversely associated with PA, particularly among subjects in the oldest age groups; this association has also been observed in previous studies [14]. The finding of an inverse association among young males between 25–44 years could be related to the beginning of the parenthood period. Having a first child significantly decreases PA levels of young adult males, but not in females because males have a comparatively greater amount of PA to lose as a result of becoming a parent [20].

The fact that educational level was directly related to the dichotomous dependent variable (i.e. < vs. ≥500 MET-minutes per week) but inversely related to the continuous dependent variable (i.e. number of MET-hours per week) could be explained by the hypothesis that the performance of additional PA, beyond minimum recommended levels, might not necessarily result in greater health benefits for an individual [21]. Thus, it could be inferred that relative to less-educated individuals, more-educated individuals have a better understanding of the minimum levels of PA that healthcare authorities recommend for health maintenance.

Concerning employment status, having an occupation was directly related to the volume of PA performed by young and middle-aged individuals. These results could be understood based on the recent study of Colman et al. [22] who found that, relative to employed subjects, unemployed subjects show increased levels of recreational PA but suffer a net reduction in total PA, due to the decrease in work-related exertion that the lack of work involves. In this sense, it would be desirable to consider individuals’ job type in future studies. Previous studies determined that “blue-collar” working-class jobs have higher work-related PA, but that this is still correlated with poorer health outcomes, and that benefit derived from both work-PA and leisure-PA is restricted to “white-collar” jobs [23]. Besides, unemployed people have lower economic status which may in turn explain reductions in PA levels, although this could not be confirmed in our models due to lack of economic data. The finding of an inverse association between having a remunerated occupation and performing minimum recommended PA levels in men of around 64 years old is clearly related to retirement.

### Implications for research and practice

Because an active lifestyle is an essential factor in the prevention of many diseases and in the management or minimisation of the progression of chronic disease processes, interventions to increase PA are essential. There is strong evidence indicating that among older adults, PA is associated with higher levels of functional health, decreased risks of falling, and improved cognitive function [1, 6, 7]. Moreover, Moschny et al. found that poor health was the most important barrier preventing adequate PA execution by older individuals [24]. From a cross-sectional perspective, our findings revealed lower PA levels among both the youngest and the oldest adults with multiple chronic conditions, which supports the need to adapt PA recommendations to the characteristics of specific population groups [25].

The drafting of these recommendations should involve appropriately qualified professionals from every relevant sector. Although evidence regarding the effectiveness of PA-related counselling interventions in the primary care setting has been inconclusive [26, 27], certain authors still view this approach as a promising means for promoting PA in adults [26]. Moreover, in 2001, the US Community Preventive Services Task Force published a report with a series of evidence-based strategies to promote PA in different communities; these strategies emphasised community-wide campaigns, school-based physical education, and social support interventions in community settings [28].

### Limitations and strengths

The main limitation of the present study relates to its cross-sectional design, which prevents us from establishing any causal inference. Longitudinal analyses are therefore required to gain further knowledge and confirm the hypotheses suggested in the present study.

Although the survey response rate was high (73.3%), the generalizability of the findings could still be biased. Increasing the sample size by means of substitution does not remove the non-response bias; it just increases the precision of obtained estimates.

The European Health Interview Survey list of chronic conditions could be considered limited, and thus may underestimate the true burden of multimorbidity. Moreover, our measure of multimorbidity was limited to the number of chronic diseases, without any assessment of disease severity. These aspects could be considered in future studies.

Another limitation is related to the measure of PA levels. Individuals were asked about their PA levels in the seven days prior to the interview which may not be representative of their general PA. Moreover, seasonal variations were not considered and this could have an influence in the amount of PA performed. Yet, this survey represents a reliable and valid information source offering data for an extensive population sample. In contrast to other PA measures with greater validity and reliability (such as accelerometers [29, 30], GPS technologies [31], and pedometers [32]), questionnaires are not only inexpensive and easy to administer but also avoid modifying individuals' behaviours [33]. One issue is that many extant surveys measuring PA are not comparable with respect to the data collection or the type of activities that are examined. In this sense, the European Health Interview Survey is based on the short version of the IPAQ, which has been validated in 12 countries and was developed to guide policy development related to health-enhancing PA in various life domains [18].

The use of the METs score as an indicator of PA intensity provides a good estimate of an individual’s self-determined gross energy expenditure. The calculation of MET-minutes per week was based on the standards of the IPAQ [18], which were set forth in response to a global demand for valid PA measures that were comparable within and between countries. Rutten et al. also used these scores to estimate volumes of PA in various European countries [34]. Moreover, the use of METs allowed us to generate a dichotomous variable based on the minimum PA threshold recommended by the ACSM, a recognised international institution. Nevertheless, this threshold must be interpreted with caution since it is simply an approximation that has been generalised to entire populations.

The socio-economic status of subjects should also have been considered to account for the potential confounding effects of this factor; however, the incorporation of this variable was not possible due to low response rates. Kaplan et al. also refrained from including this variable in their analyses, with the explanation that health behaviours are more closely associated with education than with occupation or income [14]. Other lifestyle covariates, such as alcohol and tobacco consumption, body mass index, and diet should also be included in future studies. It would also be interesting to compare our results from Spain with those from other European countries based on the same health survey.

## Conclusions

The present study demonstrated an inverse relationship between multimorbidity and PA among youngest and older adults. In addition, low self-rated health status and the presence of functional limitations are both associated with lower PA levels above the age of 45 years. These features should be considered in the design and implementation of community-based approaches to promoting PA, if further corroborated in longitudinal studies.

Future large prospective population-wide studies using standardised PA measures are required to further explore the temporality of the associations observed in this investigation and better explain why changes in the nature of the dependent variable produced different results.

## Abbreviations

PA: Physical activity
MET: Metabolic equivalent
ACSM: American College of Sports Medicine
AHA: American Heart Association
IPAQ: International Physical Activity Questionnaire
PASE: Physical Activity Scale for the Elderly.
